# Supplementary material for: Dynasore Blocks Ferroptosis through Combined Modulation of Iron Uptake and Inhibition of Mitochondrial Respiration
Source: Cells. 2020 Oct 9;9(10):2259. doi: 10.3390/cells9102259 (PMC7650611; doi:10.3390/cells9102259)
Supplement: Supplementary file 1 [file cells-09-02259-s001.zip › cells-883401-supplementary.docx]

**Supplementary Figures**

**Supplementary Fig. S1.**

**Dynasore blocks ferroptosis at various doses.**

**
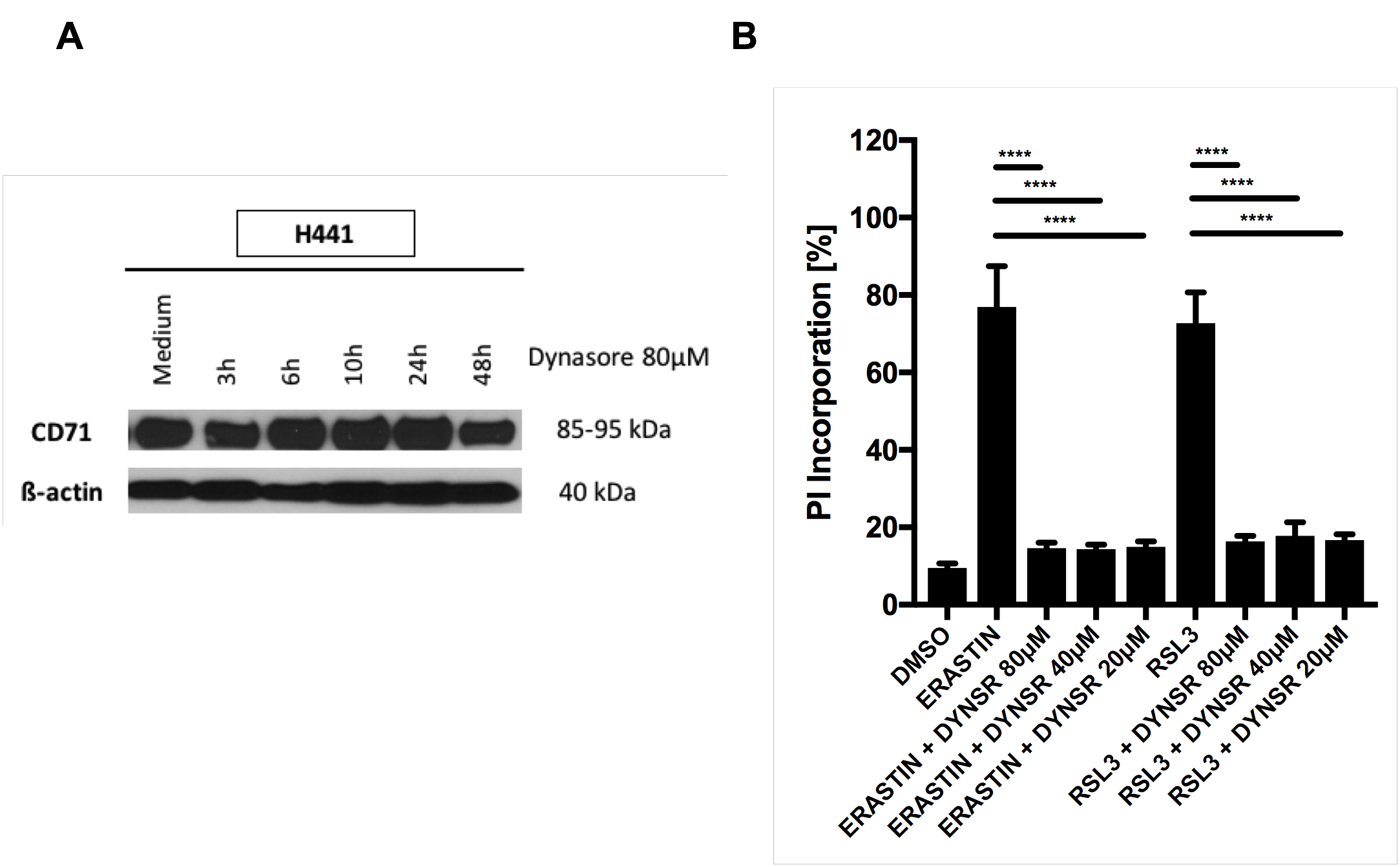
**

**Figure S1.** (**A**) Total expression levels of CD71 was determined in H441 cells after treatment as indicated by Western blotting (cropped). (**B**) H441 cells were treated with DMSO, erastin [10 µM], RSL3 [1 µM] +/- the indicated concentrations of dynasore for 48 h. Cell death was quantified by uptake of propidium iodide (PI) uptake and flow cytometry. Data are means +/- SEM of at least three independent experiments. Representative blots are shown.

**Supplementary Fig. S2.**

**Dynamins regulate iron uptake but this fails to block lipid peroxidation.**
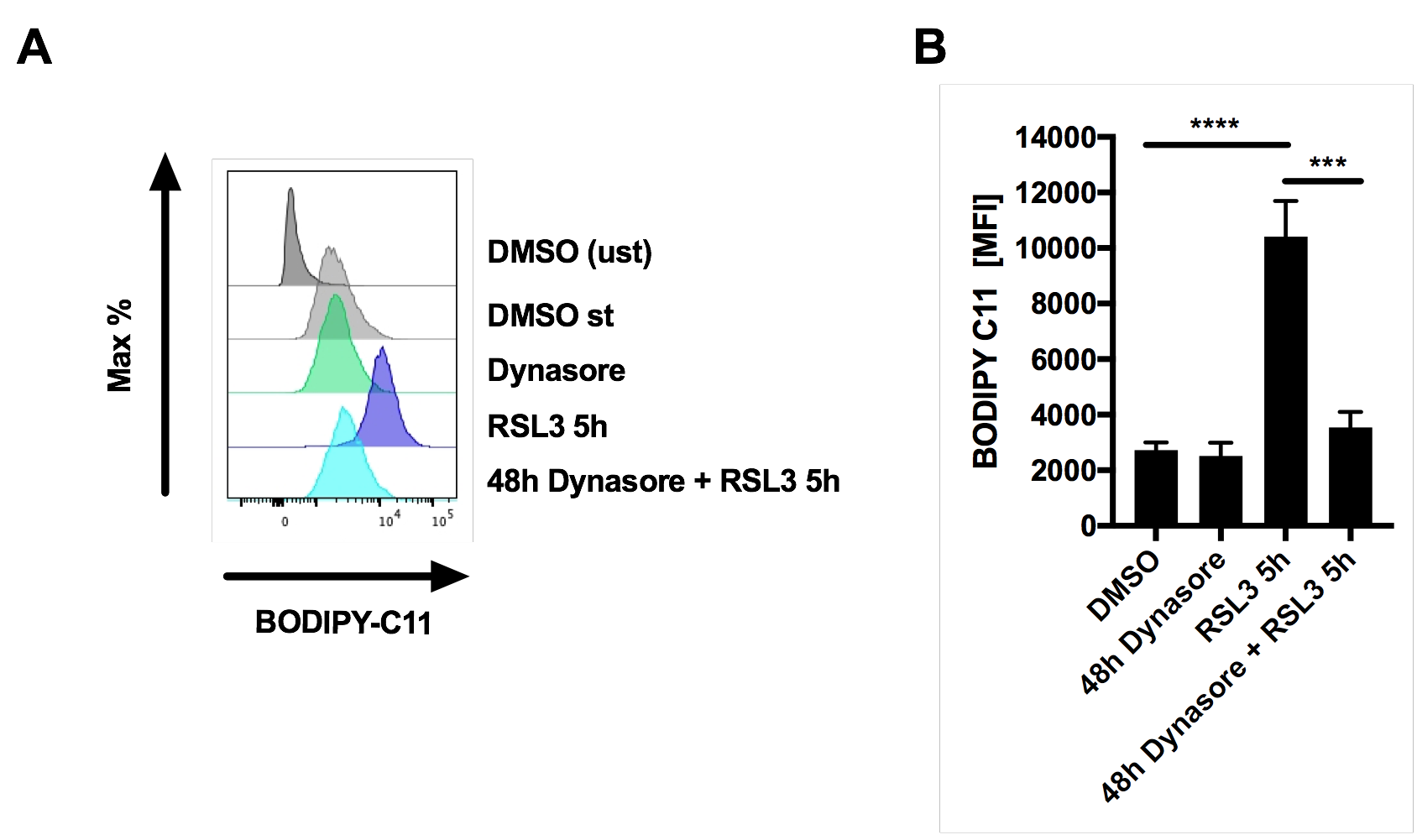


**Figure S2.** (**A**, **B**) H441 cells were treated with DMSO or dynasore [80 µM] for 48 h. RSL3 [1 µM] or DMSO were added for 5 h. During the last 30 min BODIPY C11 was added at 5 µM to each well. Mean fluorescence intensity (MFI) was quantified by flow cytometry. Data are means +/- SEM of at least three independent experiments. Representative histograms are shown.

**Supplementary Fig. S3.**

**Dynasore blocks mitochondrial ROS but does not affect mitochondrial respiration in cell-free systems.
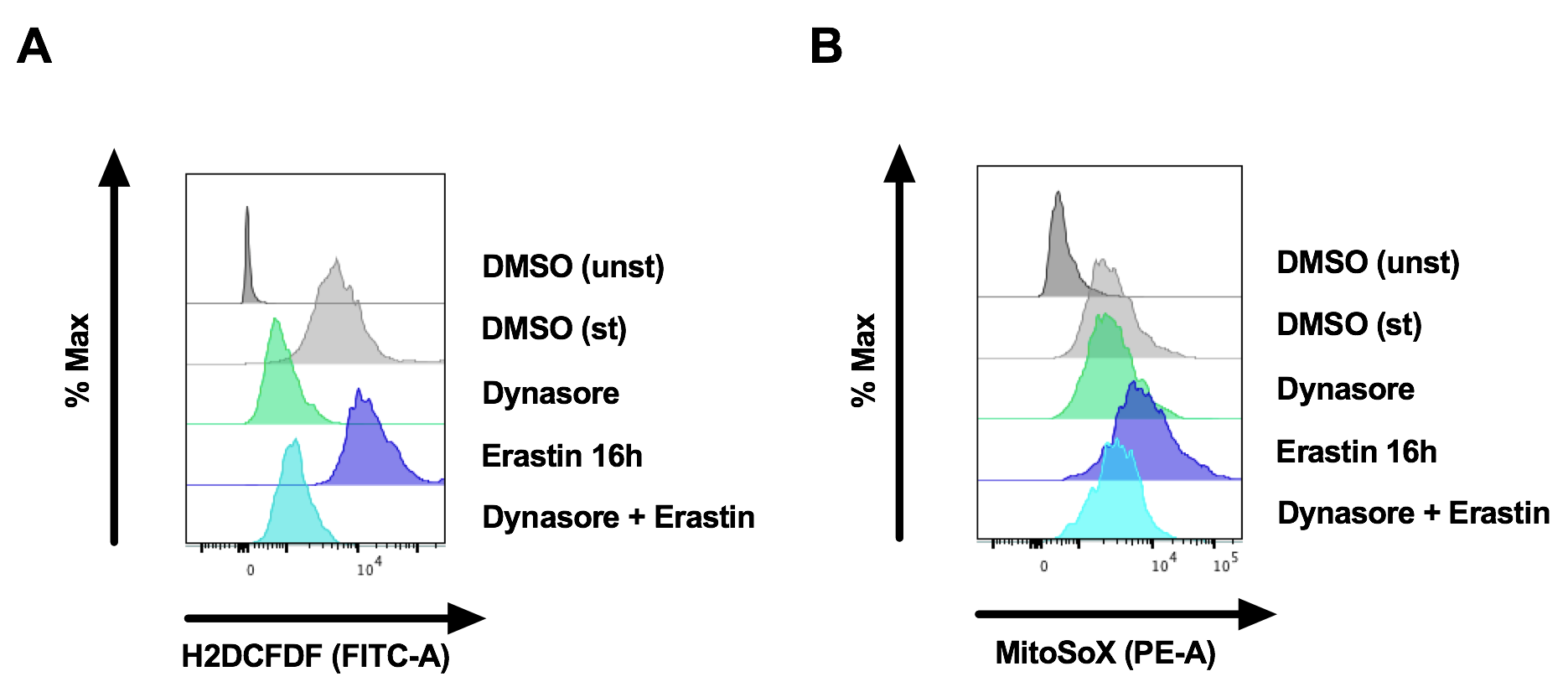
**

**Figure S3.** (**A**, **B**) H441 cells were treated with DMSO or dynasore [80 µM] for 48 h. erastin [10 µM] or DMSO were added for 16 h. During the last 30 min H2DFCFD or MitoSox were added at 20 µM and 2 µM, respectively to each well. Representative histograms are shown. Data are means +/- SEM of at least three independent experiments. Representative histograms are shown.

**Supplementary Fig. S4.** Uncropped image for Fig. 2a and Supplementary Fig. S1a.

**Related to Fig. 2a**

**
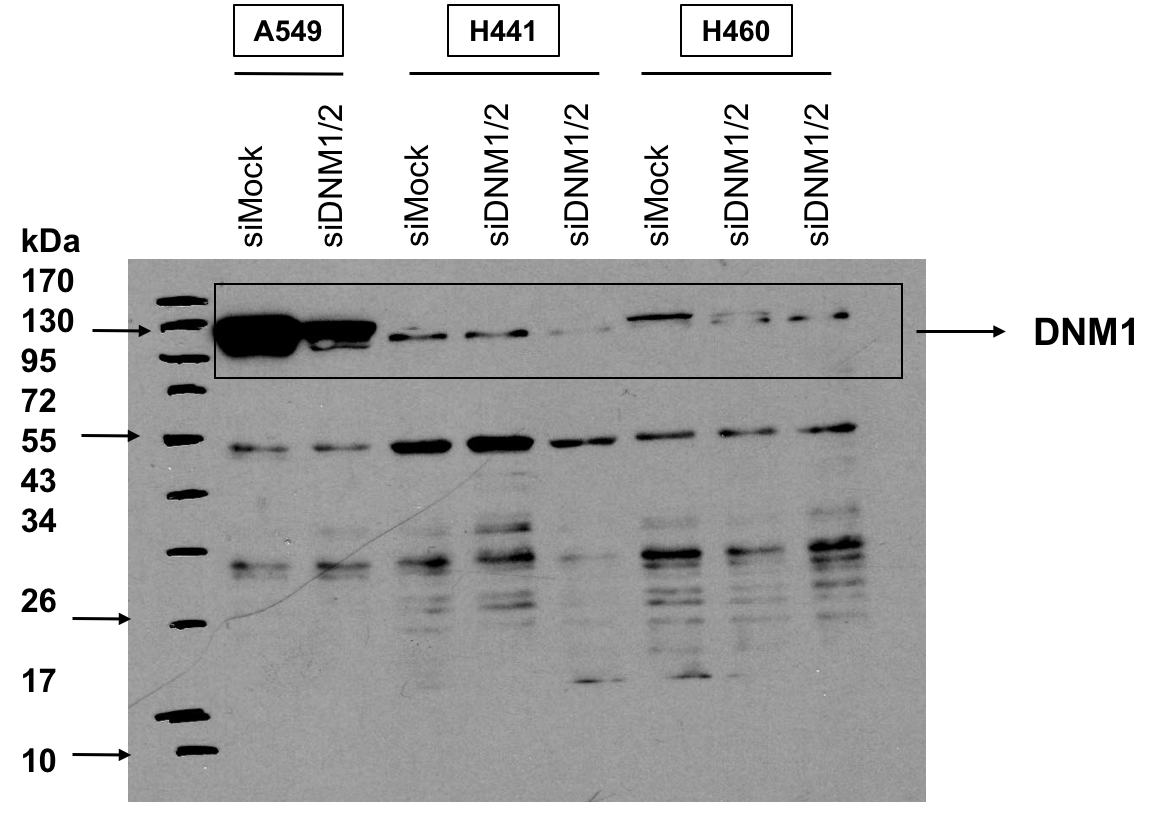
**

**
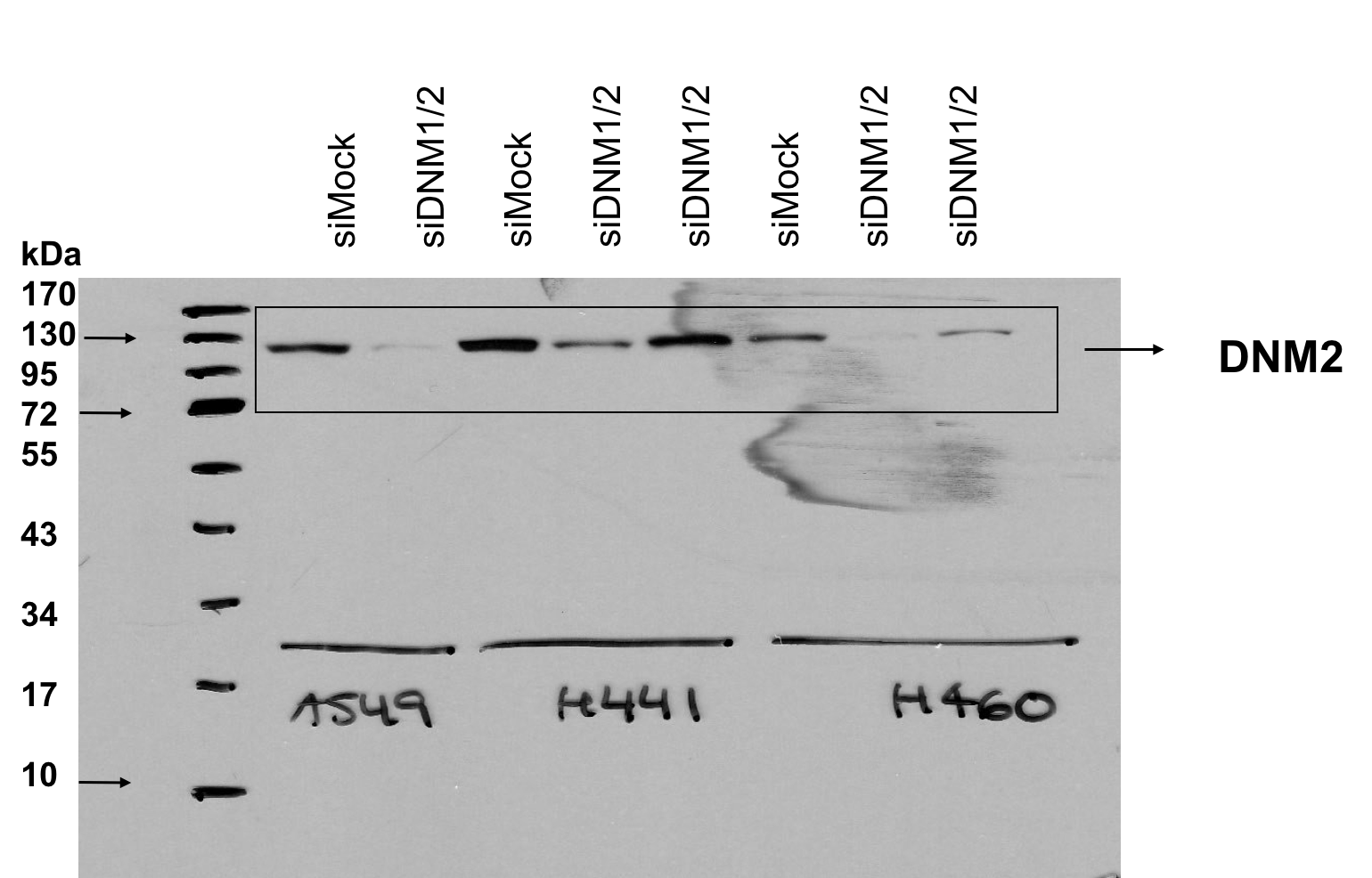
**

**
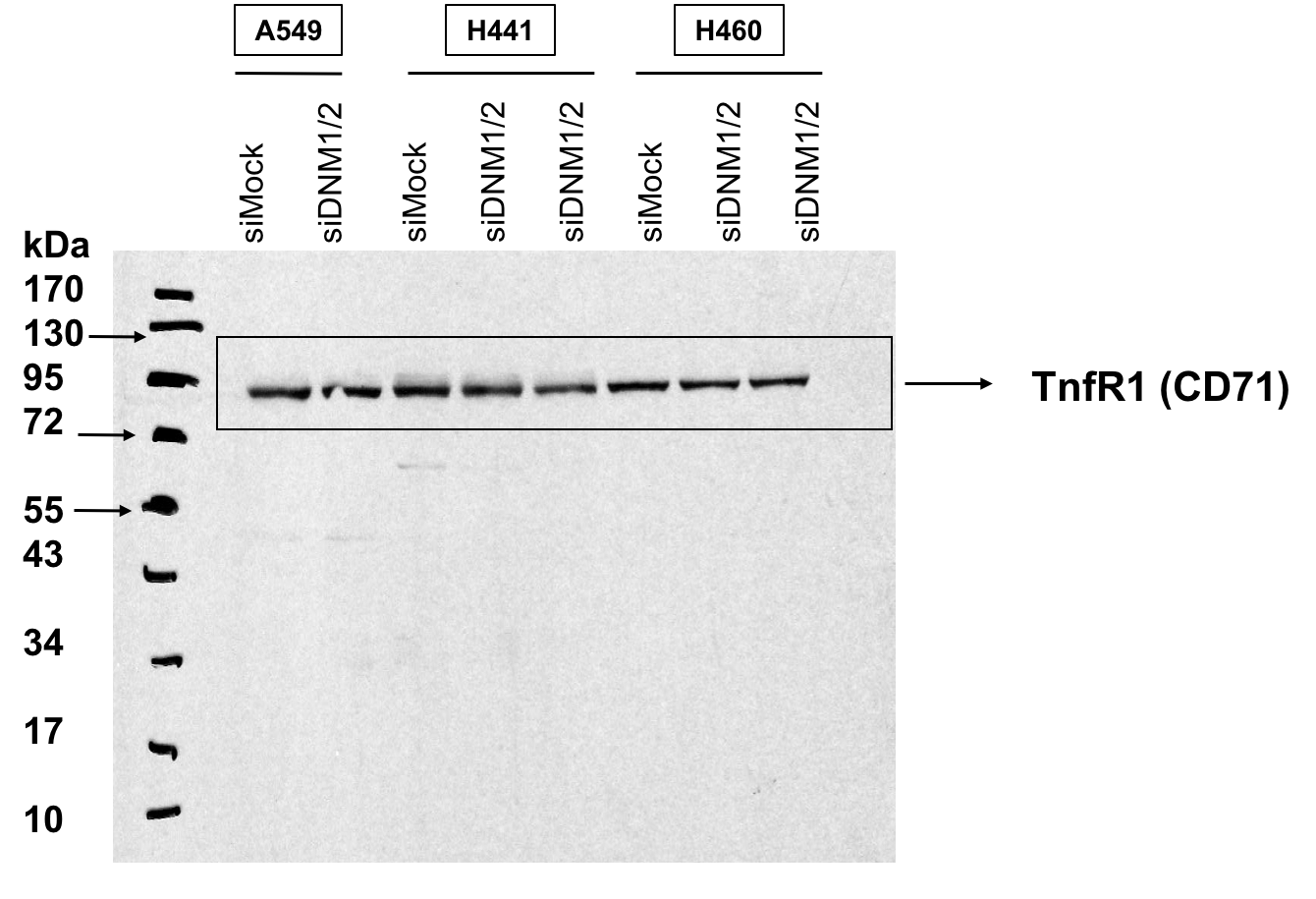
**

**
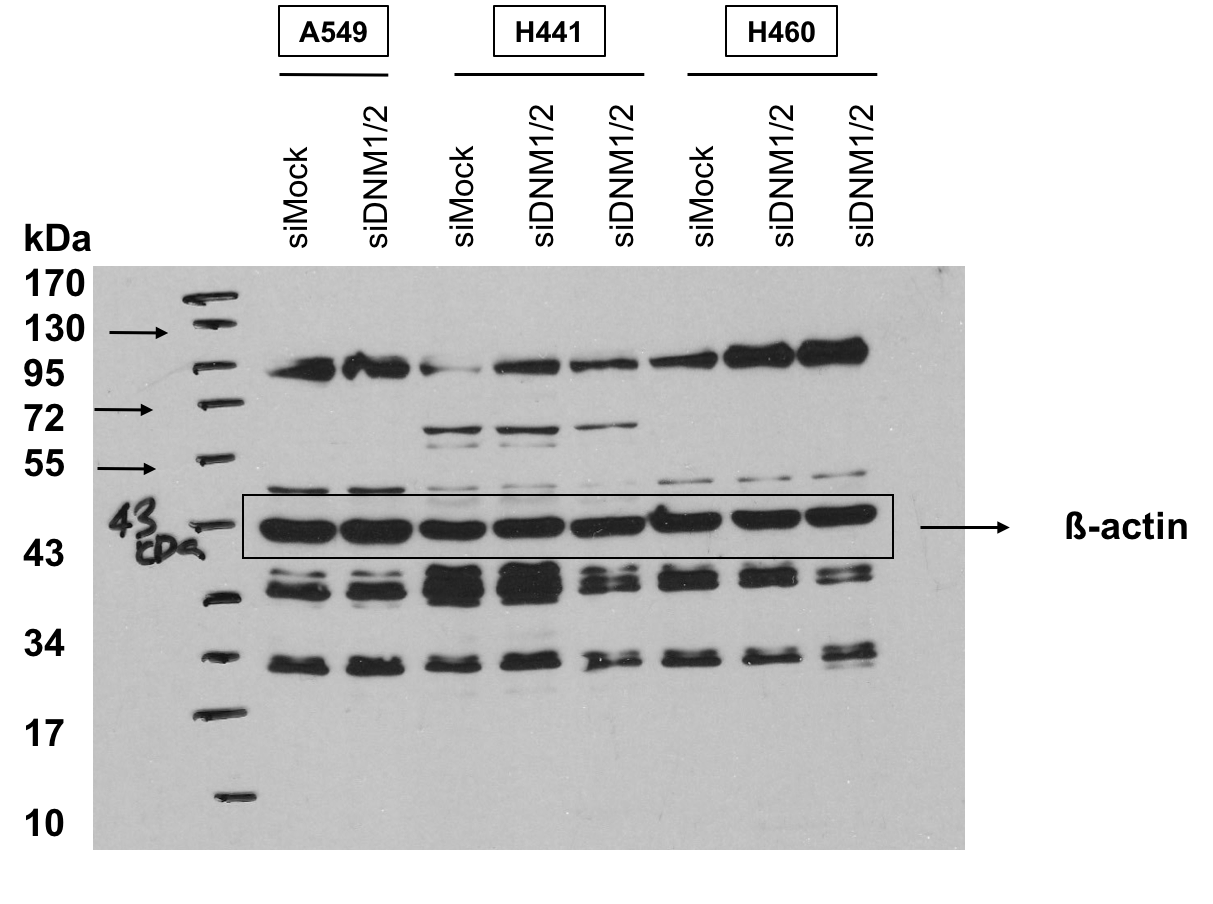
**

**Related to Supplementary Fig. S1a**

**
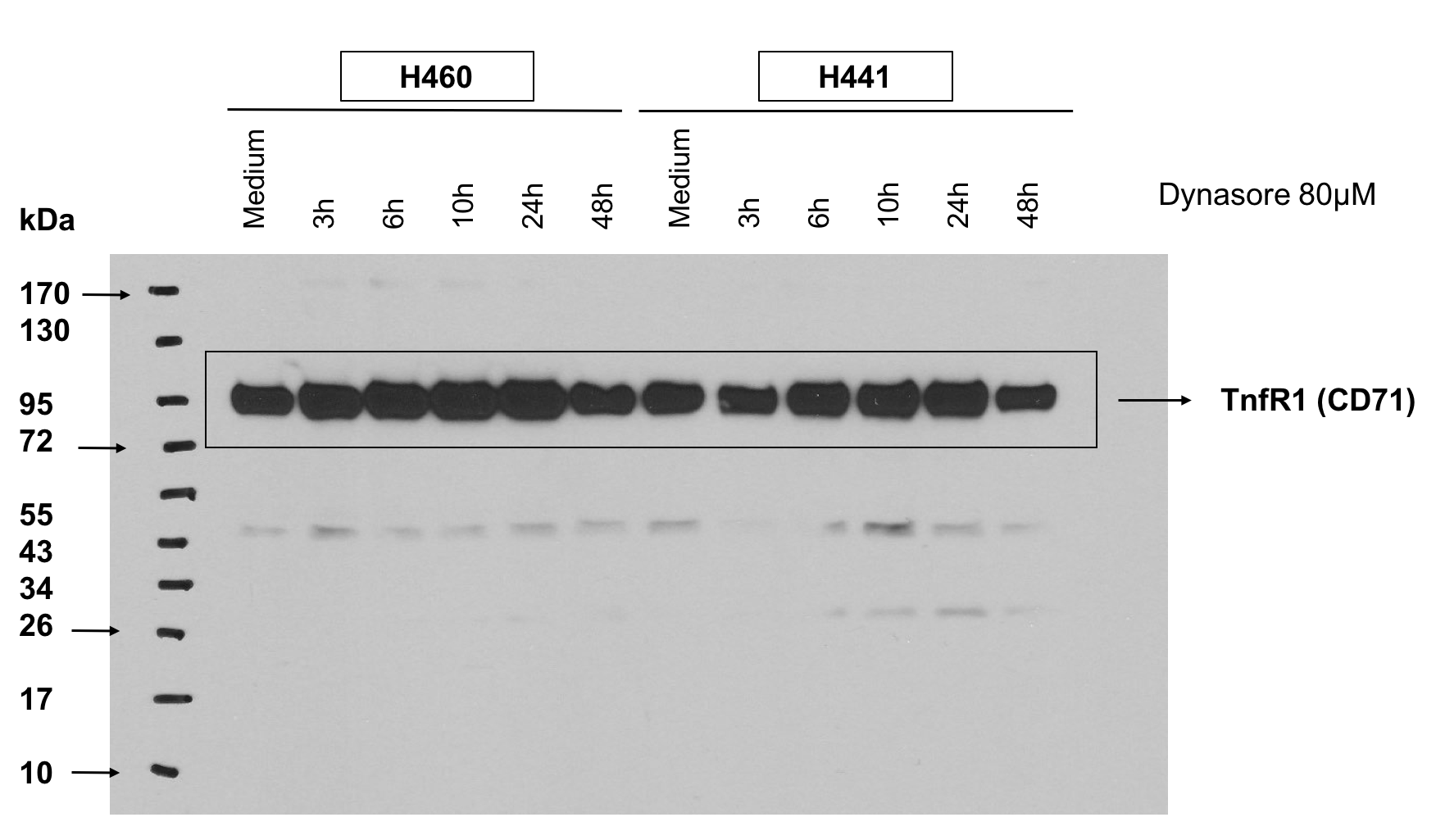
**

**
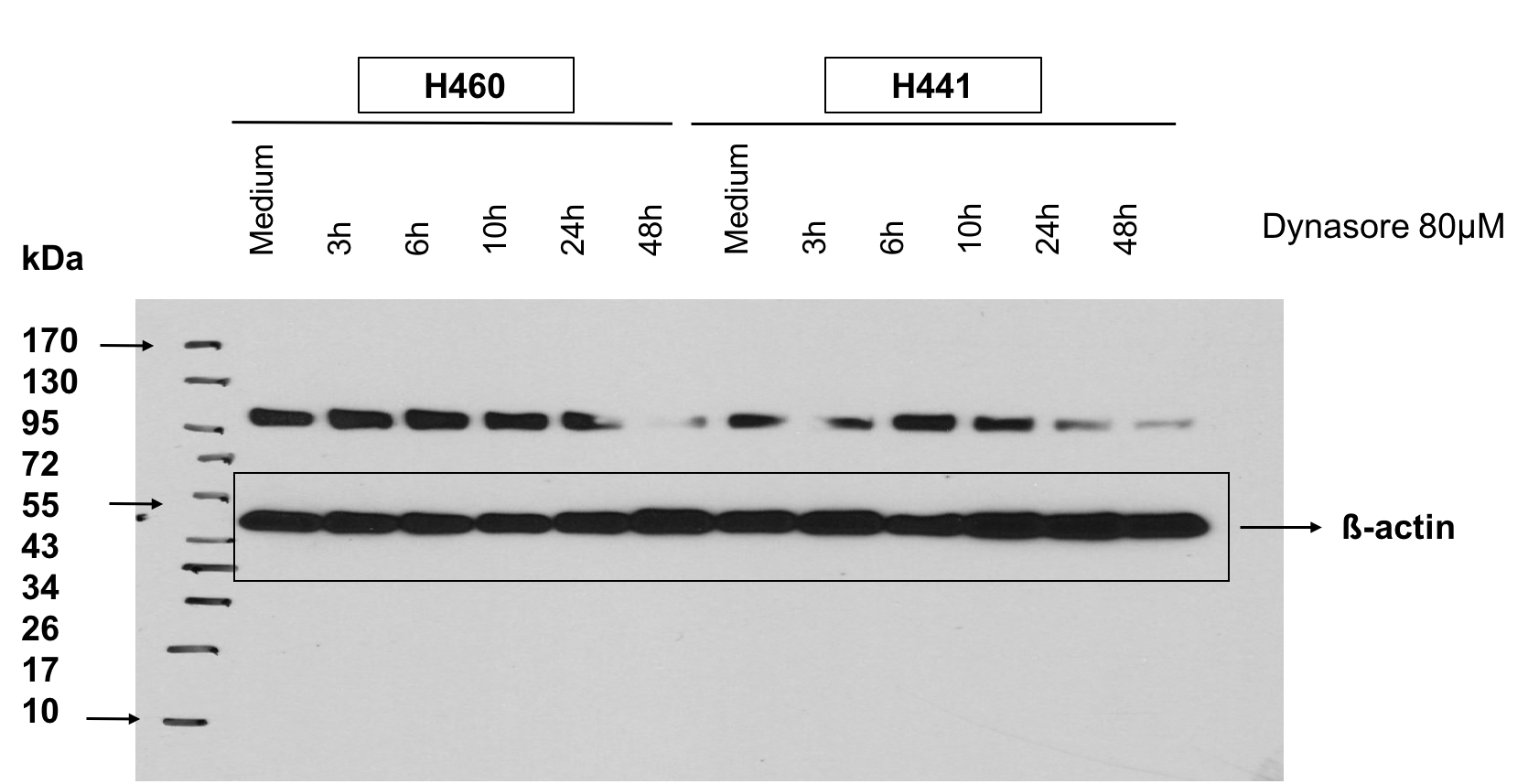
**
